# Ecological and Epidemiological Findings Associated with Zoonotic Rabies Outbreaks and Control in Moshi, Tanzania, 2017–2018

**DOI:** 10.3390/ijerph16162816

**Published:** 2019-08-07

**Authors:** Niwael Mtui-Malamsha, Raphael Sallu, Gladys R. Mahiti, Hussein Mohamed, Moses OleNeselle, Bachana Rubegwa, Emmanuel S. Swai, Selemani Makungu, Edward G. Otieno, Athuman M. Lupindu, Erick Komba, Robinson Mdegela, Justine A. Assenga, Jubilate Bernard, Walter Marandu, James Warioba, Zacharia Makondo, Jelly Chang’a, Furaha Mramba, Hezron Nonga, Japhet Killewo, Fred Kafeero, Yilma J. Makonnen, Ariel L. Rivas, Folorunso O. Fasina

**Affiliations:** 1 Food and Agriculture Organization of the United Nations, Dar es Salaam 14111, Tanzania; 2 Department of Developmental Studies, Muhimbili University of Health and Allied Sciences, Dar es Salaam 11103, Tanzania; 3 One Health Central and Eastern Africa, Dar es Salaam 11103, Tanzania; 4 Ministry of Livestock and Fisheries, Dodoma 41000, Tanzania; 5 College of Veterinary Medeicine and Biomedical Sciences, Sokoine University of Agriculture, Morogoro 67000, Tanzania; 6 One Health Coordination Desk, Prime Minister’s Office, Dodoma 41000, Tanzania; 7 Ministry of Health, Community Development, Gender, Elderly and Children, Dodoma 41000, Tanzania; 8 District Veterinary Office, Moshi District 25101, Tanzania; 9 Zonal Veterinary Center, Arusha 23000, Tanzania; 10 Tanzania Veterinary Laboratory Agency, Dar es Salaam 15101, Tanzania; 11 Food and Agriculture Organization of the United Nations, 00153 Rome, Italy; 12 Center for Global Health, School of Medicine, University of New Mexico, Albuquerque, NM 87131, USA; 13 Department of Veterinary Tropical Diseases, Faculty of Veterinary Science, University of Pretoria, Onderstepoort 0110, South Africa

**Keywords:** human-animal interaction, One Health, rural community, rabies, wildlife, zoonosis

## Abstract

Approximately 1500 people die annually due to rabies in the United Republic of Tanzania. Moshi, in the Kilimanjaro Region, reported sporadic cases of human rabies between 2017 and 2018. In response and following a One Health approach, we implemented surveillance, monitoring, as well as a mass vaccinations of domestic pets concurrently in >150 villages, achieving a 74.5% vaccination coverage (*n* = 29, 885 dogs and cats) by September 2018. As of April 2019, no single human or animal case has been recorded. We have observed a disparity between awareness and knowledge levels of community members on rabies epidemiology. Self-adherence to protective rabies vaccination in animals was poor due to the challenges of costs and distances to vaccination centers, among others. Incidence of dog bites was high and only a fraction (65%) of dog bite victims (humans) received post-exposure prophylaxis. A high proportion of unvaccinated dogs and cats and the relative intense interactions with wild dog species at interfaces were the risk factors for seropositivity to rabies virus infection in dogs. A percentage of the previously vaccinated dogs remained unimmunized and some unvaccinated dogs were seropositive. Evidence of community engagement and multi-coordinated implementation of One Health in Moshi serves as an example of best practice in tackling zoonotic diseases using multi-level government efforts. The district-level establishment of the One Health rapid response team (OHRRT), implementation of a carefully structured routine vaccination campaign, improved health education, and the implementation of barriers between domestic animals and wildlife at the interfaces are necessary to reduce the burden of rabies in Moshi and communities with similar profiles.

## 1. Introduction

Rabies is caused by a neurotropic virus of genus *Lyssavirus*, family *Rhabdoviridae*. Domestic dogs are important maintenance hosts although other carnivores may be involved [1]. Most cases of human rabies are associated with suspected rabid dog bites, through an infectious virus in their saliva and records of animal bite injury are accurate predictors of rabies exposure [2]. Approximately 59,000 human deaths, 3.7 million Disability-Adjusted Life Years (DALYs) loss and US$8.6 billion in economic losses per annum from premature deaths, associated productivity losses and post-exposure prophylaxis (PEP) exist [3].

Endemic rabies in Tanzania claims 1499 human deaths annually [4,5,6,7], and the Kilimanjaro Region (KR) had reported frequent outbreaks. Between June 2017 and March 2018, seven humans were reported bitten by suspected rabid dogs in Moshi Rural District (MRD), of which four (57%) were children. Of the affected humans, four died, two could not be traced further while one survived. Five cattle were also exposed to rabid dog bites out of which three (60%) died. Six dogs were involved but only one was owned. Importantly, Mt. Kilimanjaro has a rich eco-biogeographical diversity and is inhabited by at least 154 mammal species, some of which may act as niches for rabies virus [8]. The inhabitants of villages around the edge of the mountain live off the abundant natural resources with resultant intense human–domestic animal–wildlife interactions. To date, only domestic dogs and probably jackal and bat-eared foxes have been epizootiologically and phylogenetically suggested as alternative hosts for rabies across Africa [1,9,10,11,12]. 

MRD has approximately 509,431 humans (2017 population estimates) [13], and a combined owned and unowned/scavenging domestic dog and cat populations of 40,102. A previous report has indicated that an ecosystem like Moshi is suitable for dog movement and that ownerless free-range dogs may have a home range of 1.9 km^2^ (range: 0.2–8.5 km^2^) [14]. MRD has a total land area of 1713 km^2^ and with 26,718 dogs and 13,390 cats (2018 estimates), a dog and cat density of 16/km^2^ and 8/km^2^ were estimated respectively. Mass dog vaccination campaigns with coverage >70% are the primary control measure against rabies infection in humans and animals [15], but this vaccination coverage has hardly been attained in most developing countries [16].

Pre-exposure vaccination for high-risk groups (veterinarians and human health workers, rabies vaccinators and laboratory workers) is carried out to protect them from occupational-associated infections during manipulations of potentially infectious animals and materials and PEP is given to patients with a history of dog bites. In Tanzania, over 20% of rabies-exposed individuals do not seek medical treatment and are not documented in official records, while less than 65% received PEP [3]. To date, the prevailing situation, detailed surveillance methods, diagnostic strategies, elimination, and control methods have been detailed in the Guidelines for Surveillance of Prioritized Zoonotic Diseases for Human and Animal Health in the United Republic of Tanzania (URT) [17], and the URT National Rabies Control Strategy [18].

The objectives of the current study are to (a) understand dog owners’ perceptions, knowledge of host susceptibility, transmission, and control measures for rabies; (b) determine the seroprevalence of rabies infection and estimate risk factors for rabies seropositivity in dogs in Moshi.

## 2. Materials and Methods

### 2.1. Study Area

MRD is one of the six districts in KR. Administratively, the district has 4 divisions, 31 wards, and over 150 officially registered villages. The study was carried out between April and May 2018, but vaccination continued until September 2018. The KR has an estimated human population of 1,640,084, the majority of which are involved in crop cultivation and livestock rearing. The study was part of an emergency response to rabies outbreaks in MRD supported by the FAO Component of the USAID funded “Supporting Global Health Security Agenda (GHSA) to address Zoonotic Diseases and Animal Health in Africa (GHSA-ZDAH)” project. KR represents the rabies endemic area with the highest dog bites records in Tanzania. The sample size of 286 dogs, mostly non-descript, and of all ages and sex was estimated based on an animal-level prevalence of 25%, and 95% certainty of detection using the Sample Size for a Proportion analytic tool in OpenEpi Version 3.01 (Rollins School of Public Health, Atlanta, GA, USA) [19]. A total of 342 dogs were sampled from the four divisions; however, a representative proportion of the wards and villages including the numbers of samples per division were conveniently selected randomly based on the total number of dogs presented for vaccination per location, which was not easy to determine from the onset as dogs and cats were presented randomly from every village.

### 2.2. Questionnaire Design and Data Collection

Data collecting tools (semi-structured questionnaire, focus group discussion (FDG) and key informant’s interview (KII)) were developed and deployed to gather the required information (supplementary material). Pre-testing of the questionnaire was undertaken with five experts. To ease data processing, minimize variation, and improve response precision, more of the closed-ended (categorical) questions were used.

FGDs, guided by a checklist, was administered to dog owners and KII were conducted for livestock extension officers, village leaders, the Regional Medical Officer (RMO), the District Medical Officer (DMO) and the DVO. The number of participants for FGDs ranged from 6–7/site. Data collection was conducted by expert social scientists and continued until the saturation point was reached (no new issue was raised by participants). Taking representative samples of each village in the four divisions, a total of 215 persons were conveniently sampled until the end of the exercise.

### 2.3. Sample Collection

Sterile sampling materials were used to collect saliva and sera from randomly selected dogs and were kept on ice (−4 °C). All samples were dispatched to the Centre for Infectious Diseases and Biotechnology (CIDB), Tanzania Veterinary Laboratory Agency (TVLA), Dar es Salaam and kept at −81 °C (or −20 °C) until analyzed.

### 2.4. Ethical Clearance

Written consent forms were provided to all participants and only those who signed the consent form participated in the interviews. Interviewed participants were informed that they reserved the right to discontinue participation if they so wish. The Moshi District Council and the Directorate of Veterinary Services, Ministry of Livestock and Fisheries, United Republic of Tanzania provided permission to obtain and process samples through the approval numbers: MDC/V/10/3/84 and PA.116/340/01 respectively.

### 2.5. Laboratory Analysis

In total, 278 sera collected from the campaign and submitted to the CIDB-TVLA were tested for rabies diagnosis. The serum samples, stored at −20 °C, were utilized for blocking-ELISA as per ^®^BioPro ELISA Antibody protocol (O.K. SERVIS BioPro, Bořetická, Czech Republic). The investigated sera were diluted to half of the sample diluent (60 µl + 60 µl) in a dummy plate. Positive and negative control sera were diluted similar to the test sera. Control sera were vortexed before dispensing followed by incubation with biotinylated anti-rabies antibody and with Streptavidin conjugate at 37 ± 2 °C for 30 min, with gentle shaking on an orbital shaker. This was followed by incubation with TMB substrate for 15–30 min at room temperature (18–25 °C) and gentle shaking away from direct sunlight. The reaction was stopped by dispensing 50 µl of stop solution per well. Optical density (OD) was read at 450 nm. Validation was based on the kit protocol and the panel of control sera were used for internal quality control and test optimization. The results were interpreted by calculating the percentage of blocking (PB) as per the manufacturer’s instructions (serum sample with PB lower than 40% = negative and PB ≥ 40% = positive for rabies antibodies).

### 2.6. Data Analysis

All data were filtered and checked for consistencies. Human (ownership) and dog-level data including ELISA serology results were matched on Microsoft Excel^®^ (version 2013, Microsoft Corporation, Redmond, WA, USA) by two independent researchers and confirmed by a third person for accuracy. Descriptive statistics for the dog and owner-level explanatory variables examined in the study were developed using Microsoft Excel^®^ (version 2013, Microsoft Corporation, Redmond, WA, USA) statistical package.

Epidemiological data were transformed and coded for Stata v 9.0. (Stata Corp., College Station, TX, USA). Independent variables were tested for pairwise associations, using a two-tailed chi-square test. Relationships between explanatory (independent) variables (owner and dog level) and outcome variables (seroconversion status: negative/positive) were investigated in two steps by logistic regression. In the first step, relationships between each independent and outcome variable were individually investigated. In the second step, any variables that were significantly associated at the *p* < 0.3 level were included in the multivariable model. A backward selection procedure was applied using a selection threshold of *p* ≤ 0.05 to reduce the number of variables in the model. All the excluded variables were then individually re-tested in the model and retained if they were significant with the Hosmer–Lemeshow goodness of fit *χ*^2^; the Akaike information criterion (AIC) and the Bayesian information criterion (BIC) were conducted to check the model fit.

## 3. Results

### 3.1. Study Demographics (Human and Animal)

A total of 215 interviews were conducted with 100% response rate, with 8% (4.3–11.5%) of all respondents were female. Overall, the age of respondents ranged from 15–89 years, median = 36 years, but the age of household head ranged from 19–90 years, median = 50 years. The average household size was five (*n* = 185) and the majority of the respondents (62.8%) have primary school levels of education (Table 1). Whereas respondents may sometimes own up to eight dogs, the average number of dogs per household was two dogs and in the case where cats are owned, an additional two cats were present on average (Table 1). Only 14.9% of all respondents’ households owned cats.

### 3.2. Rabies Awareness and Knowledge among Respondents

Most of the respondents (94.4%) were aware of rabies and 89.8% indicated that rabies affects animals, but only 43.9% knew that the disease affects multiple species (dogs, cats, cattle, sheep, goats, and humans) (Table 2). On further investigation, only 55.8% were able to indicate with clarity, signs of rabies in domestic animals (change of behavior–aggression, barking, hydrophobia, pica, fever, seizures, paralysis, dropped jaw, inability to swallow, and change in barking tone (Table 2)). Vaccination of dogs and cats against rabies were carried out by only 37.4% of the respondents (Table 2). However, the rabies vaccination statuses of 34.2% (*n* = 27) have lapsed between 2014 and 2016 and just 62.0% (*n* = 49) have up-to-date vaccinations (2017), and 3.8% cannot remember when the vaccination was carried out. Only one cat was declared vaccinated in 2017. Of the 215 respondents, 78.6% indicated that rabies affected humans and on average only 42.3% could indicate clearly the observed signs of rabies in humans (mental confusion, altered consciousness with history of animal bite). Approximately 75.4% were aware of the modes of rabies transmission in humans and animals, and 15.4% indicated that at least a family member had been bitten by a suspected rabid dog, or affected by rabies in other ways (7.4%, Table 2).

### 3.3. Dog Bites Victims and Post-Exposure Prophylaxis

From the 33 individuals previously bitten by dogs or whose family members were bitten, 60.6% indicated that dog-bite victims should mandatorily wash fresh wounds thoroughly, be taken to a hospital and report incidents of dog bites to authorities. Of the 33 respondents, 75.8% indicated receipt of PEP injection by self or family victims, and only 72.7% survived while 15% died with no details on the remaining four individuals. With regards to non-family victims of dog bites, 39.4% indicated knowledge of receipt of PEP injection by a non-family victim, and only 58.3% have taken correct actions following dog bites; in addition, 9.1% indicated that the bite victims died due to complications possibly associated with dog bites (Table 2).

### 3.4. Laboratory Findings

Only 278 of 342 sera collected were correctly matched with epidemiological details, hence all follow-up analyses were based on 278 samples. The remaining 64 cryovials were either not matched epidemiologically (*n* = 18) or contained empty samples/missing labels (*n* = 46). A seropositivity rate of 33.8% was obtained based on c-ELISA results (Table 3). For risk analysis, the seropositive dogs were subdivided into vaccinated (31.7%, *n* = 88) and unvaccinated (68.3%, *n* = 190). These results were further categorized into the following: (i) vaccinated and seropositive animals (34/278); (ii) vaccinated and seronegative animals (54/278); (iii) non-vaccinated but seropositive animals (60/278); and (iv) non-vaccinated and seronegative animals (130/278).

### 3.5. Secondary Data

**Figure 1:**
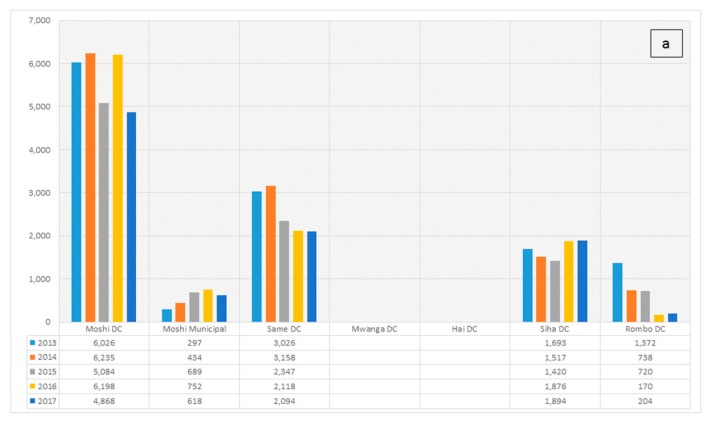

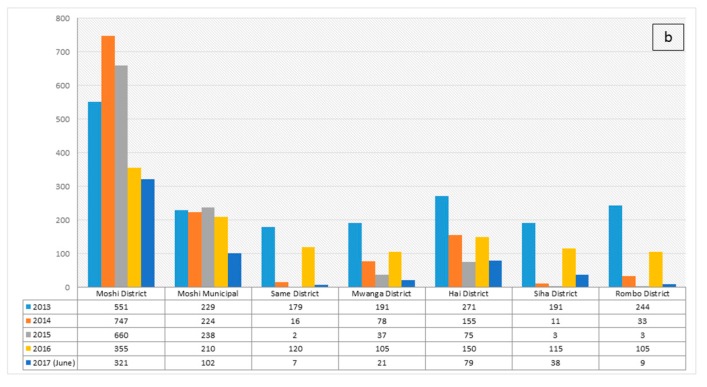
(**a**) Rabies vaccination in the Kilimanjaro Region, 2013–2017; (**b**) Incidence of dog bites reported in the Kilimanjaro Region, 2013–2017. Note that the incidence of dog bites is not indicative of the total number of rabies cases. Dog bites can be associated with many sources of aggression, like the provocation of dogs, entering the dog territories, possessiveness, response to a painful injury, fear, maternal instinct, pursuant of prey, rabies-associated aggression among others.

Dog vaccination records and human PEP reports collected from the DVO and DMO Offices and Regional Veterinary Office indicated that in 2017, MRD veterinary office had vaccinated 4781 dogs (≈ 18% dog-level vaccination coverage) and 168 persons received PEP in the last 15 months. Rabies vaccination statuses of other districts in the KR were indicated below (Figure 1a). In terms of dog bites, MRD reported the highest number of dog bites (45% of the total 5875 human victims) over the period covering 2013–2017 (Figure 1b). The estimated incidence of dog bite cases was 83 cases per 100,000 human population using the data from 2012–2017. Between 2013 and 2016, a total of 33 samples from suspected rabid cases and none was confirmed positive. However, in 2017, of the ten samples submitted, five (two humans and three cattle, with a history of dog bites) were confirmed with direct fluorescent antibody technique. As of April 2018, eight samples were received and six were confirmed positive including four from humans and two from cattle.

### 3.6. Factors Influencing Seroprevalence to Rabies Virus Infection

#### 3.6.1. Univariate and Multivariate Analysis

The association of the owner- and dog-level categorical explanatory variables at *p* ≤ 0.30 and Rabies virus infection serostatus is shown in Table 4a. Twenty-one risk variables were individually analyzed and only 11 qualified for inclusion in the multivariable analysis. The included variables are outlined in Table 4a. In the final multivariable logistic regression model, only two factors were retained (strongly associated with increased odds of rabies seropositivity) including: Dogs and cats are more than 50% of the household livestock populations (OR = 2.24; 95% CI = 1.17–4.28; *p* = 0.01) and dogs and cats were observed to have interacted with and mixed with wildlife (OR = 3.62; 95% CI = 1.00–13.13; *p* = 0.05), Table 4b. 

#### 3.6.2. Qualitative Findings

Twenty FGDs and thirteen KII were administered and poor levels of compliance to animal vaccination and a general lack of awareness were observed. Many of the interviewed individuals and groups willingly opted for vaccination but the major constraints mentioned were the non-affordability of rabies vaccines and professional services costs; and, sometimes, dubious vaccination statuses claims were given to unsuspecting officials. There was a structured annual district dog/cat vaccination schedule and the community members were aware, yet they avoided the schedules due to costs. Over 60% also avoided vaccinations due to distance from the DVO and other vaccination centers. The community agreed that the availability of large numbers of stray dogs in the community increases the risk of transmission of rabies. Sometimes, political interference enhances non-compliance by dog/cat owners; stray dogs have been seen to interact with wild animals, especially at interfaces, and <10% of the community believed that witchcraft was responsible for poor rabies control and dog bites in humans.

## 4. Discussion

Rabies has significant public health impacts in Tanzania [4,10,21]. Moshi’s record of incidence of dog bites (83/100,000 humans) may not be indicative of total number of rabies cases (as bites can be associated with other sources of aggression (provocation of dogs, entering the dog territories, possessiveness, response to a painful injury, fear, maternal instinct, pursuant of prey, and rabies-associated aggression, among others), yet it is of great public health concern because the rabies statuses of the dogs that inflicted bite injuries were unknown; in previous studies, more than 98% of rabies-related human deaths were associated with bites from rabid dogs [4,22,23,24]. This bite incidence rate in humans was lower than 140/100,000 bites reported in another study in rural Tanzania [2], and it is likely that incidences of dog bites and associated rabies cases are underreported in MRD. Anecdotal evidence and records from district hospital and veterinary offices, and highlights from the FGD indicated data gaps. Some of the bite victims obtained the initial doses of PEP but later became untraceable for follow-ups. In Haiti, only 1/3 of victims who received PEP were fully compliant with follow-ups [25].

Education level may influence the knowledge of rabies [26,27], and this was observed in Moshi. A proportion of the dogs and cats owned in the household were brought for vaccination accompanied by children ≤16 years. This confirms previous observation that children and young adult interact more and are at higher risk of exposure to rabies [28]. It becomes pertinent to increase awareness of rabies in children and young adults in order to reduce the risk of rabies through different fora including schools, community engagements, and playground using easy to adopt infographics. There was an average of two dogs per households and two cats in some instances; the presence of these animals in the households, when left unvaccinated presents a risk to other warm-blooded species, and humans in the households. Studies undertaken in Kenya and Nigeria have confirmed similar estimates per household [29,30,31].

The witchcraft association with rabies in MRD may lead to seeking spiritual assistance rather than hospitalization following dog bites and this complicates rabies epidemiology in Tanzania. Activation of the district-level One Health Rapid Response Team (OHRRT) should help minimize such data gaps.

While some districts in the Kilimanjaro region have conducted annual dog/cat vaccination, the recommended coverage remains unreached. For instance, the MRD veterinary office vaccinated 18% of dogs in 2017, a gross under-coverage, given the population of susceptible animals in this area (dogs, cats, and unknown numbers of wild canids). Regular animal vaccination program for high-risk regions should be implemented because of abundant domestic and wildlife resources that exist in this region, the public health threats associated with inaction or under-delivery in this regard. Though there is stiff competition for scarce resources in low-income countries like Tanzania, the adverse impact of the disease and high eco-tourism potentials in KR should allow public reinvestment of proportions of the revenue generated from ecotourism to combat rabies through vaccine provision and effective diagnosis. A sustained intensified awareness campaign is necessary and a pre-emptive national vaccine stockpile is warranted in these regions.

During our interviews, 79 households’ dogs and cats were vaccinated, but only 49 have up-to-date vaccination records. Those with lapsed and undated vaccinations (*n* = 30) may be predisposed to a false sense of “protected dogs and cats”, whereas these animals are potential hosts of rabies virus and may transmit the same to humans. In addition, a lack of knowledge of rabies risks and transmission, vaccination costs, and distances to vaccination centers may facilitate low vaccination compliance.

Serologically, the categories: vaccinated-seropositive and non-vaccinated-seronegative dogs are potentially safe and portend a lower risk of rabies transmission to humans; however, the vaccinated-seronegative and the non-vaccinated-seropositive dogs present a significant risk of zoonotic rabies. This finding emphasizes the need for careful reevaluation of the vaccines used for animal immunization at district levels. Adherence to comprehensive protocols on vaccination should ensure potency and immunogenicity of vaccines used in rural communities. For example, in the USAID-funded, FAO-facilitated multi-sectoral vaccination campaign conducted in 2018, the source of the vaccine was certified by the OIE, cold chain was maintained throughout, and professionals/paraprofessionals were used in the delivery of vaccines that provided coverage to 74.5% of all dogs and cats in the community [32]. Since the implementation (April 2018–March 2019), no single case of rabies has been reported in humans or animals in Moshi by the public health and veterinary authorities [33]. Furthermore, in previous studies, other factors have been linked to idiosyncratic reactions and atypical antibody responses including the following: (a): situations where temporality and longevity of humoral responses vary across individuals [34]; (b) abortive rabies infection [35]; and (c) previous predominant cellular response inhibiting humoral response [36]. Similar observations to findings from our study have also been presented by other researchers [37,38,39].

Where dog and cat proportions are higher than 50% of households’ livestock populations excluding poultry, and with intense interaction and mixing with wildlife, the odds of rabies seropositivity increased. Unvaccinated herds of maintenance host and potentials scavenging practices will enhance the risk of mixing with wildlife and these factors support exposure to rabies infection. In addition, it is likely that our sampling of dog/cat populations was biased towards places or locations where these interactions may occur most frequently because human and dog population density in such villages near to wildlife parks may increase significantly due to eco-tourism potentials. Efforts are needed at interface locations to reduce wildlife-domestic animal interactions through park fencing, animal restraint at the households, intense vaccination, and other disease control programs.

In this study, certain limitations were observed: (1) A total of 286 animals was the sample size and 342 samples were collected, yet only 278 samples were useful for risk analysis. Approximately 18.7% of the samples collected were lost due to poor labeling or leaked samples. It is important to make adequate preparation for field sampling prior to fieldwork in the future. Perhaps the use of permanent indelible markers and high-quality sample collection tubes would significantly minimize such sample loss and boost sample adequacy. Despite this limitation, the total samples were fairly representative of the divisions, wards, and villages in Moshi. (2) Although the total number of animals to be sampled were also known (*n* = 286), sampling was only structured to the divisions and only a subset of the wards and villages were sampled conveniently. It should be known that in a multi-aim complex campaign of vaccination, awareness, biosecurity messaging, and eco-epidemiologic studies of this nature, the undetermined numbers of samples that may arise from each village will add to the complexities of such a campaign, yet we made efforts to minimize this error by a relatively good sample spread. (3) While it appeared that the interviewed gender was skewed against the female and the young ones, it reflected the proportion of the household heads. It is understood that in most African communities, women and young ones remain in the background with regards to opinions on household matters. Whether this influenced our responses here is not immediately known but more balanced gender and youth representations may produce different outcomes in future studies.

## 5. Conclusions

Given the low annual vaccination coverage reported, the presence of stray dogs, the high number of victims of animal bites and the interfaces, there is a need to sustain community sensitization regarding rabies and associated control measures. Other studies [40] reported that large-scale dog vaccination campaigns and other control efforts reduced the incidence of human rabies. Similarly, a 98% reduction in rabies cases in dogs from 2005 to 2015 in the western hemisphere, contributed to a 96% reduction in human rabies [41,42]. Finally, the implementation of issues identified in the established guidelines and strategies for Tanzania, including but not limited to routine vaccination of susceptible species, is of utmost importance.

## Figures and Tables

**Table 1:** Demography of respondents and animals owned.

**Demography of Respondents**				
**Variable (*n*)**	**Category (*n*)**	**Percentage ± SE**		**95% CI**
Gender of the respondents (215)	Male (198)	92.0 ± 1.8		88.5–95.7
	Female (17)	8.0 ± 1.8		4.3–11.5
Gender of the head of household (215)	Male (197)	91.7 ± 1.9		88.0–95.5
	Female (18)	8.3 ± 1.9		4.5–12.0
Level of education (215)	No formal education (7)	3.3 ± 1.2		0.9–5.8
	Up to Primary (135)	62.8 ± 3.3		56.3–69.3
	Up to Secondary (58)	26.7 ± 3.1		20.6–32.7
	Up to Tertiary (15)	7.1 ± 1.8		3.6–10.7
	**Median**	**Mean ± SE**		**(Min, Max)**
Age Respondent (215)	36	39.3 ± 1.2		36.9; 41.6
Age Household head (202)	50	51.6 ± 1.1		49.4; 53.8
Total household size (185)	5	5.5 ± 1.9		5.2; 5.9
**Description of Owned Animals**				
**Variable (*n*)**	**Mean ± SE**	**95% CI**	**Median**	**(Min, Max)**
Dogs per household (211)	2.0 ± 0.1	1.8–2.2	1	1; 8
Cats per household (32)	1.7 ± 0.2	1.3–2.2	1	1; 7
Pigs per household (37)	4.9 ± 0.7	3.5–6.3	3	1; 15
Goats per household (96)	6.1 ± 0.8	4.5–7.7	4	1; 53
Sheep per household (26)	6.5 ± 1.9	2.6–10.3	4	1; 50
Cattle per household (109)	3.1 ± 0.3	2.4–3.7	2	1; 30
Chickens per household *	18.5 ± 2.8	13.0–24.0	11	2; 120
Dogs and cats combined per household	2.3 ± 0.1	2.0–2.5	2	1; 14

Confidence intervals at 95% (95%CI) were calculated using the binomial Wald method. * Note that an insignificant number of other poultry and rabbits also exist in the households in Moshi Rural District. 38 households received new births of dogs within the last one year, 22 brought in more dogs without health certification and five received dogs as gifts. 91.86% of 221 respondents are sedentary mixed farmers while 8.14% are agro-pastoralists.

**Table 2:** Awareness and knowledge of rabies epidemiology and transmission and control in animals and humans.

Variable	Number	Proportion ± SE	95% CI
*Animal variables*			
Awareness of rabies in animals	215	94.4 ± 1.6	91.3–97.5
Rabies affects animals	215	89.8 ± 2.1	85.7–93.9
Know rabies sign in animals	215	55.8 ± 3.4	49.1–62.5
Own animals affected	215	14.9 ± 2.4	10.1–19.7
Rabies vaccination conducted	211	37.4 ± 3.3	30.9–44.0
Human variables			
Rabies affect humans	215	78.6 ± 2.8	73.1–84.1
Know rabies sign in humans	215	42.3 ± 3.4	35.7–49.0
Aware of transmission in humans and animals	215	75.4 ± 3.0	69.5–81.2
Family members have been bitten by a suspected rabid dog	214	15.4 ± 2.5	10.5–20.3
Family members have been affected by rabies	215	7.4 ± 1.8	3.9–11.0
*Aware of family member bitten by a suspected rabid dog*			
Post-dog bite actions taken was correct	33	60.6 ± 8.6	43.0–78.2
PEP injection received	33	75.8 ± 7.9	60.3–91.9
Patient recovered	33	72.7 ± 7.9	56.7–88.8
Patient succumbed (died)	33	15.2 ± 6.3	2.2–28.1
Other animals affected	33	3.0 ± 3.0	−3.1–9.2
*Aware of non-family member bitten by a suspected rabid dog*			
Aware of another person bitten by a suspected rabid dog	33	39.4 ± 8.6	21.8–57.0
Post-dog bite actions taken was correct	33	58.3 ± 14.9	25.6–91.1
PEP injection received	33	24.2 ± 7.6	8.8–39.7
Patient recovered	33	24.2 ± 7.6	8.8–39.7
Patient succumbed (died)	33	9.1 ± 5.1	−1.3–19.4
Reported incidence	33	12.1 ± 5.8	0.4–23.9

**Table 3:** Prevalence (with exact ± 95% confidence intervals) of rabies antibodies in dogs based on c-ELISA.

Variables	Positive (%)	95% CI (%)	Negative (%)	95% CI (%)
Total sample (*n* = 278)	94 (33.8)	28.5–39.6	184 (66.2)	60.4–71.5
Vaccination history				
Yes (*n* = 88; 31.7%)	34 (38.6)	29.1–49.1	54 (61.4)	50.9–70.9
No (*n* = 190; 68.3%)	60 (31.6)	25.4–38.5	130 (68.4)	61.5–74.6
Potentially risky dogs (60 + 54)/278	114 (41.0)	35.4–46.9		

Risky groups are defined as vaccinated dogs without active immunity and non-vaccinated dog with positive serology. Confidence intervals of 95% were calculated using a modified binomial Wald method [20].

**Table 4:** (**a**) Association of antibody to rabies virus infection positives and explanatory variables (*p* ≤ 0.3) at dog, owner, and ecological level, Moshi, Tanzania. (**b**) The final logistic regression models for rabies virus infection by dog, owner, and ecological level, Moshi, Tanzania.

(**a**)				
**Variable**	**Category**	**Odds Ratio**	**95% CI**	***p*-Value**
Gender/sex of the dog	Female versus male	0.71	0.37–1.37	0.30
Age of animal	Young versus adult (over 6 months)	1.65	0.33–8.19	0.54
Park/Game reserve available in the vicinity	Available versus not available	0.64	0.28–1.46	0.29
Proportion of dogs and cats in the household livestock population	<50% versus ≥50%	1.71	0.92–3.18	0.09
New dog or cats #	No new introduction versus New introduction	0.84	0.45–1.58	0.59
Livestock observed mixed with wildlife ^1^	No versus Yes	2.73	0.80–9.34	0.11
Total household population (humans)	>5 persons versus ≤5 persons	1.76	0.88–3.48	0.11
Sighted wild animals in the vicinity	No versus Yes	0.89	0.45–1.78	0.75
Level of education (head of household)	Secondary or above versus Up to primary	0.71	0.38–1.33	0.28
Shelter for dogs at night	No versus Yes	1.46	0.66–3.24	0.35
Household members aware of rabies	No versus Yes	1.10	0.27–4.41	0.89
Household members have knowledge of rabies	No versus Yes	2.14	0.69–6.67	0.19
Animals in the household previously affected by rabies	No versus Yes	0.87	0.39–1.95	0.73
Know that rabies affects humans	No versus Yes	1.01	0.49–2.08	0.99
Household member was previously affected by rabies	No versus Yes	0.31	0.07–1.40	0.13
Aware of transmission of rabies	No versus Yes	0.77	0.38–1.56	0.47
Household member was previously bitten by rabies	No versus Yes	0.49	0.17–1.38	0.18
Aware of community member bitten by a dog	No versus Yes	1.04	0.50–2.15	0.92
Report previous incidence known	No versus Yes	1.07	0.31–3.71	0.91
Observed dog roaming	No versus Yes	1.93	0.78–4.77	0.15
Own dog scavenged	No versus Yes	2.13	0.85–5.33	0.11
(**b**)				
Variable *	**Z-Score**	**Odds Ratio**	**95% CI**	***p*-Value**
Dogs and cats are more than 50% of the household livestock population	2.45	2.24	1.17–4.28	0.01
Livestock (dog & cats) observed to mix with wildlife	1.96	3.62	1.00–13.13	0.05

(**a**) 95% CI = 95% Confidence interval; # new dogs or cats were introduced through births, new purchases or gifts; ^1^ Wildlife sighted includes the following: fox, hyena, komba, squirrel (vicheche), leopard, mangrove, wild dogs, monkeys, impala (swala), warthog, and rabbits. (**b**) Hosmer-Lemeshow Goodness of fit χ^2^ = 6.96; *p*-value = 0.92; AIC (Akaike Information Criterion) = 245.13; BIC (Bayesian Information Criterion) = 254.84. * Offset variable was a combination of eco-epidemiological variables: (Vicinity to National park, Forest Reserve, Game Reserve, Game-controlled Area, and River Reserve).

## Supplementary Materials

The following are available online at https://www.mdpi.com/1660-4601/16/16/2816/s1, Rabies Household Questionnaire.
