# Supplementary material for: Ecological and Epidemiological Findings Associated with Zoonotic Rabies Outbreaks and Control in Moshi, Tanzania, 2017–2018
Source: Int J Environ Res Public Health. 2019 Aug 7;16(16):2816. doi: 10.3390/ijerph16162816 (PMC6719226; doi:10.3390/ijerph16162816)
Supplement: Supplementary file 1 [file ijerph-16-02816-s001.pdf]

RABIES HOUSEHOLD QUESTIONNAIRE

Name of Enumerator………………………………………….. Date……………………District…Moshi…………………Village……….............. ....

Vaccination centre………………………

A: DEMOGRAPY

GPS coordinates Lat: …………. Long: …………. Elevation (m) ………….

1. Household data

i.	Name of the Respondent		ii.	Age<br>Gender *(tick√)*	Yrs..………….<br>M….F….
iii.	Head of the household	Age (yrs.) ………….<br>Gender (tick√) M….F…	iv.	Number of people living in the household	M…………<br>F…………
v.	Level of Education (tick√)	No formal education<br>Completed Primary school<br>Completed Secondary school<br>Tertiary<br>Other (specify)………………………...			

B: HERD DYNAMICS

2. Herd size: how many animals of different species and ages are in the herd?

Species	Young (<6 months for dogs, < I year for pigs, goats, sheep and <2 years cattle and camels)		Mature (≥6 months for dogs, ≥I year for pigs, goats, sheep and ≥ 2years cattle and camels)		Total
	Male	Female	Male	Female	
i. Dogs					
ii. Cats					
iii. Pigs					
iv. Goats					
v. Sheep					
vi. Cattle					
vii. Other (specify)…… ……					

3. In the past year, how many animals were added to the herd and reasons for addition?

Species	No. born	No. bought	No. gifts received or borrowed	Other reasons for additions
i. Dogs				
ii. Cats				
iii. Pigs				
iv. Goats				

RABIES HOUSEHOLD QUESTIONNAIRE

Name of Enumerator………………………………………….. Date……………………District…Moshi…………………Village……….......... ....

Vaccination centre……………………

v. Sheep				
vi. Cattle				
vii. Other (specify)… ………				

4. In the past year, how many animals have left the herd and reasons for leaving?

Species	No. died	No. sold	No. slaughtered	No. gifted or loaned	No. Stolen	No left due to Predation	Other (specify)
i. Dogs							
ii. Cats							
iii. Pigs							
iv. Goats							
v. Sheep							
vi. Cattle							
vii. Other (specify) …………							

5. What were the reasons for death(s), if any in the herd *(tick√)*?
   i. Old age
   ii. Still birth
   iii. Predation
   iv. Drought
   v. Disease (specify)...
   vi. Unknown
   vii. Other (specify)...

6. What is the main reason for keeping each species?

Species	Dairy	Meat	Drought power	Security	Other (specify)
Cats					
Dogs					
Pigs					

RABIES HOUSEHOLD QUESTIONNAIRE

Name of Enumerator…………………………………………..
Date……………………District…Moshi.…………………Village………..........
....
Vaccination centre……………………

Goats					
Sheep					
Cattle					
Other (specify)………………….					

7. What is the main farming system? *(tick√)*
   i. Sedentary mixed farming
   ii. Agro-pastoralist
   iii. Pastoralist

8. Housing: are the livestock enclosed? *(tick√)*
   Yes (   )   No (   )

9. If Yes, how are they kept? *(tick√)*
   i. Enclosed at night
   ii. Enclosed during day
   iii. Enclosed during day and night

10. Do you provide shelter for your dog? *(tick√)*
    Yes (   )   No (   )

C: CONTACTS WITH WILDLIFE

11. Over the past 12 months have you seen wild animals near your village? *(tick√)*
    Yes (   )   No (   )

12. If Yes, what type of wild animals did you observe?
    i. Wild dogs (mbwa mwitu)
    ii. Hyena (fisi)
    iii. Fox (mbweha)

    ----

13. Interaction with wildlife: do your animals often mix with wildlife? *(tick√)* Yes (   ) No (   )
14. If Yes, where do they commonly interact? *(tick√)*

Species	During grazing	At watering point	During scavenging	Other activity
Cats				

RABIES HOUSEHOLD QUESTIONNAIRE

Name of Enumerator…………………………………………..
**Date……………………District…Moshi…………………Village………............
...........**
Vaccination centre……………………

Dogs				
Pigs				
Goats				
Sheep				
Cattle				
Other (specify)				

15. Presence of wildlife ecosystem in the village: which wildlife ecosystem present nearby the village?

Wildlife ecosystem	Name	Approximate distance from village
National Park		
Forest Reserve		
Game Reserve		
Game-controlled Area		
Other		

D: KNOWLEDGE OF ANIMAL DISEASES

16. What are the diseases that you commonly experience and name animal type affected?

i……………………………………………

ii……………………………………………

iii……………………………………………

iv……………………………………………

v……………………………………………

17. Are there any animals in the herd that are currently sick? *(tick√)* Yes/No

18. Describe the clinical signs of the disease if they currently have any sick animals

**Animal type affected**	**Suspected disease**	**Local disease name**	**Number affected**	**Main clinical signs** *(loss of appetite, weight loss, swollen lymph nodes, red urine, changed behaviour …….)*	**Occurred last year (Yes/No)**

RABIES HOUSEHOLD QUESTIONNAIRE

Name of Enumerator…………………………………………..
Date……………………District…Moshi…………………Village………….............
....
Vaccination centre……………………

19. When was the last time you vaccinated your animals?

**Type of animal vaccinated**	**Disease**	**When vaccinated**
i. Dogs		
ii. Cats		
iii. Pigs		
iv. Goats		
v. Sheep		
vi. Cattle		
vii. Other (specify).........................		

E: GENERAL KNOWLEDGE ON RABIES

20. Are you aware of diseases called Rabies? *(tick√)* Yes (   ) No (   )
21. Does rabies affect animals? *(tick√)* Yes (   ) No
22. What animals do the diseases affect? *(tick√)*
    Dogs (   ) cats (   ) sheep (   ) goats (   ) cattle(   ) others (specify)......
23. Has any of your animals affected by rabies? *(tick√)* Yes (   ) No (   )
24. Does the disease affect human too? *(tick√)* Yes (   ) No (   )
25. Has any member of your family affected by rabies? *(tick√)* Yes (   ) No (   )
26. Mention any 3 signs of Rabies in animals and humans

	human	animals
i.		
ii.		
iii.		

27. Do you know how rabies is transmitted to animals or humans? *(tick√)* Yes (   ) No (   )
28. In the last 12 months has anyone in your family been bitten by a dog? *(tick√)* Yes (   ) No (   )
29. If Yes, what did you do about the bite? *(tick√)*
    i. Nothing (   )
    ii. Washed/bandage (   )

RABIES HOUSEHOLD QUESTIONNAIRE

Name of Enumerator…………………………………………..

Date……………………District…Moshi…………………Village………...................

Vaccination centre………………………

iii. Used herb ( )

iv. Other ………………………………......

30. Were you given any injections? *(tick√)* Yes ( ) No ( )
31. What type of injection?
    i. tetanus
    ii. rabies
    iii. I don't know
32. Did the affected person recover *(tick√)* Yes ( ) No ( )
33. If No, did the person succumb to rabies *(tick√)* Yes ( ) No ( )
34. Is there any animal in your house that was affected? *(tick√)* Yes ( ) No ( )
35. Do you know of any other person in your communities bitten by a dog in the last 12 months *(tick√)* Yes ( ) No ( )
36. If Yes, what did they do about the bite? *(tick√)*
    v. Nothing ( )
    vi. Washed/bandage ( )
    vii. Used herb ( )
    viii. Other ………………………………......
37. Was the victim given any injections? *(tick√)* Yes ( ) No ( )
38. What type of injection?
    iv. tetanus
    v. rabies
    vi. I don't know
39. Did the affected person recover *(tick√)* Yes ( ) No ( )
40. If No, did the person succumb to rabies *(tick√)* Yes ( ) No ( )

F: TRACEABILITY, OTHER SOURCES OF INFORMATION

Key Informants/Local Leaders/

1. Any report of the similar incident within the locality? *(tick√)* Yes ( ) No ( )
2. If yes where……………………………………………………...

OBSERVATION

Presence of dogs not housed, roaming in the village

Scavenging practices
